# The ventilatory response to hypoxia is blunted in some preterm infants during the second year of life

**DOI:** 10.3389/fped.2022.974643

**Published:** 2022-10-26

**Authors:** Zoe Freislich, Benjamin Stoecklin, Naomi Hemy, J. Jane Pillow, Graham L. Hall, Andrew C. Wilson, Shannon J. Simpson

**Affiliations:** ^1^ Wal-yan Respiratory Centre, Telethon Kids Institute, Perth, Australia; ^2^ Department of Neonatology, University Children's Hospital Basel UKBB, Basel, Switzerland; ^3^ School of Human Sciences, The University of Western Australia, Perth, Australia; ^4^ Curtin School of Allied Health, Curtin University, Perth, Australia; ^5^ Department of Respiratory and Sleep Medicine, Perth Children's Hospital, Perth, Australia

**Keywords:** hypoxia, infant, premature, bronchopulmonary dysplasia, respiration, artificial

## Abstract

**Background:**

Preterm birth and subsequent neonatal ventilatory treatment disrupts development of the hypoxic ventilatory response (HVR). An attenuated HVR has been identified in preterm neonates, however it is unknown whether the attenuation persists into the second year of life. We investigated the HVR at 12–15 months corrected postnatal age and assessed predictors of a blunted HVR in those born very preterm (<32 weeks gestation).

**Methods:**

HVR was measured in infants born very preterm. Hypoxia was induced with a three-step reduction in their fraction of inspired oxygen (F_I_O_2_) from 0.21 to 0.14. Respiratory frequency (*f*), tidal volume (*V*_T_), minute ventilation (*V*_E_), inspiratory time (*t*_I_), expiratory time (*t*_E_), *V*_T_/*t*_I_, t_I_/*t*_TOT_, *V*_T_/*t*_TOT_, area under the low-volume loop and peak tidal expiratory flow (PTEF) were measured at the first and third minute of each F_I_O_2_. The change in respiratory variables over time was assessed using a repeated measures ANOVA with Greenhouse-Geisser correction. A blunted HVR was defined as a <10% rise in *V*_E_, from normoxia. The relationship between neonatal factors and the magnitude of HVR was assessed using Spearman correlation.

**Results:**

Thirty nine infants born very preterm demonstrated a mean (SD) HVR of 11.4 (10.1)% (increase in *V*_E_) in response to decreasing F_I_O_2_ from 0.21 to 0.14. However, 17 infants (44%) failed to increase *V*_E_ by ≥10% (range −14% to 9%) and were considered to have a blunted response to hypoxia. Males had a smaller HVR than females [Δ*V*_E_ (−9.1%; −15.4, −2.8; *p* = 0.007)].

**Conclusion:**

Infants surviving very preterm birth have an attenuated ventilatory response to hypoxia that persists into the second year of life, especially in males.

## Introduction

The hypoxic ventilatory response (HVR) is a peripheral respiratory chemoreflex, stimulated by a fall in the arterial partial pressure of oxygen (PaO_2_) (1, 2). The peripheral respiratory chemoreceptors display a degree of plasticity in oxygen sensing during the perinatal period (3, 4). At birth, the infant transitions from a relatively hypoxic *in utero* environment to the normoxic ex utero environment (4). During this transition, peripheral chemoreceptors “reset” to the higher PaO_2_, becoming more sensitive to PaO_2_ levels below the new baseline. The normal biphasic HVR of neonates is characterised by an initial increase in minute ventilation due to increases in both tidal volume and respiratory rate (augmentation phase), followed by a decrease to pre-hypoxic levels or lower, due to reductions in respiratory rate (depressive phase) (5). The depressive phase may result from a reduction in metabolic rate (6, 7), as well as immature mechanisms of the respiratory control center (8). However, while a reduction in metabolic rate during hypoxia is common in newborn mammals, there has been conjecture about this phenomenon in infants (9). In contrast to the neonatal HVR, the mature chemoreflex displays a longer period of increased minute ventilation (between 10 and 20 min), followed by a smaller decrease in minute ventilation. Moreover, the minute ventilation remains higher than pre-hypoxic levels (for at least the length of a 25 min hypoxia exposure) (10).

The maturation of the HVR continues through infancy. Several previous studies in both preterm and term infants (9, 11–17) have investigated the postnatal maturation of the HVR from an immature biphasic response to the sustained hyperpnoea seen in adults. However, the age at which the infant HVR becomes similar to the adult response remains controversial. Some studies report an established “adult” response by 10 days of life (11). While others report that the immature biphasic response persists into the second month of postnatal life (12), and even at 5–6 months of age term infants fail to exhibit a sustained hyperpnoea during hypoxia (18). Development of the HVR appears similar in healthy preterm (with later gestation and no neonatal supplemental oxygen or mechanical ventilation) and term infants (19). However, the most preterm neonates often suffer from intermittent or chronic hypoxia and hyperoxia due to an immature respiratory drive, underlying respiratory disease and the associated neonatal intensive care unit (NICU) treatments (19). Fluctuations between hypoxic and hyperoxic states potentially disrupt the postnatal development of the HVR (20, 21). Shorter gestation and low birthweights of the preterm neonate predict a dampened post-natal maturation of the HVR (22, 23). Additionally, infants with evolving bronchopulmonary dysplasia (BPD) have a markedly reduced or absent HVR in the first weeks of postnatal life (24, 25). Furthermore, infants with extended oxygen therapy experience a blunted HVR up to 14 weeks post-natal age (23). Emerging data suggest potential long-term alteration in cardiopulmonary control for those born preterm (19, 26). However, it is unknown whether long-term development of the HVR is hampered by increased duration of oxygen therapy and respiratory support such as mechanical ventilation or continuous positive airway pressure (CPAP) in the NICU.

We aimed to explore the hypoxic ventilatory response to a stepwise reduction in the fraction of inspired oxygen (F_I_O_2_) in preterm born infants at 12–15 months corrected postnatal age. Further, we aimed to determine if the magnitude of the HVR beyond the first year of life was associated with neonatal factors, such as duration of supplemental oxygen and duration of respiratory support. We hypothesised that preterm infants would mount a HVR at 12–15 months postnatal age, but that the HVR would be blunted (defined *a priori* a*s* <10% increase in minute ventilation) in those receiving prolonged oxygen therapy and/or respiratory support, during the neonatal period.

## Methods

### Study population

Infants enrolled in the study were born very preterm [<32 weeks gestational age (GA)] at King Edward Memorial Hospital with no congenital abnormality. All infants were part of the Preterm Infant Functional and Clinical Outcome (PIFCO) study, a cohort evaluating the pulmonary and cardiovascular outcomes following preterm birth (ACTRN12613001062718). Neonatal clinical information and data on respiratory support were obtained from the prospectively collected PIFCO REDCap database (27, 28). Bronchopulmonary dysplasia was defined as supplemental oxygen for more than 28 days after birth, as per the 2001 National Institute of Child Health and Development (NICHD) diagnostic criteria (29). This paper focuses on the ventilatory measurements recorded in a subset of the PIFCO cohort that underwent infant lung function tests at Princess Margaret Hospital, Perth during the 12–15 month follow-up. The PIFCO follow up study was approved by the WA Princess Margaret Hospital (PMH) Human Research Ethics Committee (HREC reference number: 2014083EP), and informed written and verbal consent was obtained from the parent(s) prior to the measurements.

### Study protocol

Infants were sedated with 80 mg/kg chloral hydrate (orally) for the duration of the infant lung function testing. The infant's level of consciousness and capillary refill time were measured every 15 min by the attending physician. Heart rate and peripheral oxyhaemoglobin saturation (SpO_2_) (MasimoSET® Radical-7™, Masimo Corporation, French's Forest/NSW) were measured continuously. The dynamic oxygen test was performed at the end of the infant's lung function test session, approximately 40 min after the infant fell asleep.

### Dynamic oxygen test

**Figure 1:**
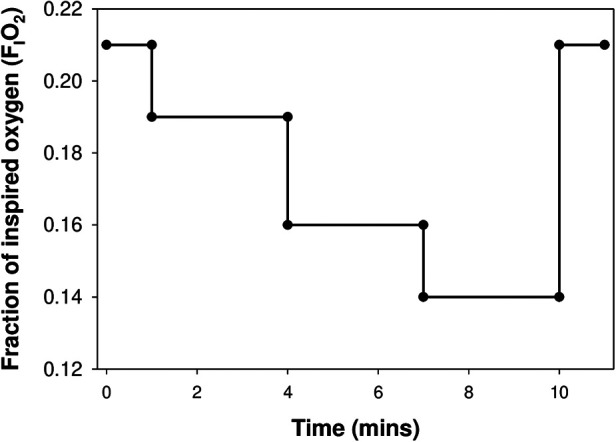
The testing protocol: inspired oxygen was reduced in 3 steps from 21% (baseline) to 14%, with each step lasting for 3 min, before the infant was returned to room air.

The F_I_O_2_ was reduced stepwise from baseline (0.21 O_2_, room air) to 0.19, then 0.16 and finally 0.14 oxygen in nitrogen balance, delivered at a continuous flow of 10 L/min *via* a sealed face mask (Figure 1). Equilibration of the oxygen mixture was instantaneous within the face mask, as continuously measured by an O_2_/CO_2_ analyser (Respiratory Gas Analyzer, ML206, ADInstruments, New Zealand), sampled *via* a port proximal to the facemask. The respiratory gases, F_I_O_2_ and SpO_2_ were recorded continuously and then analysed offline using Lab Chart (Lab Chart 7 pro, version 7.3.4, ADInstruments, New Zealand). Each step lasted for 3 min duration, with tidal breathing measured during the first (to assess the peak acute response to the reduction in F_I_O_2_), and third minute of each F_I_O_2_ step using an ultrasonic flowmeter (Exhalyzer D, EcoMedics, Duernten, Switzerland).. Ventilation variables, including inspiratory time (*t*_I_), expiratory time (*t*_E_), total breath duration (*t*_TOT_), time to peak tidal expiratory flow (PTEF), tidal volume (*V*_T_), respiratory rate (*f*), minute ventilation (*V*_E_), *V*_T_/*t*_TOT_, *V*_T_/*t*_I_, *t*_I_/*t*_TOT_ and area of the flow-volume loop were determined (WBreath version 3.19.60, EcoMedics, Duernten, Switzerland).The change in ventilatory variables (Δ) from baseline were determined by subtracting baseline measurement from those obtained during the first and third minute of each F_I_O_2_ step.

### Statistical analysis

Differences in neonatal, demographic and HVR characteristics between preterm born infants with and without a diagnosis of BPD were assessed using Student's T- test or Mann-Whitney U test as appropriate for the data distribution. Change in each tidal breathing variable over time was assessed using repeated measures ANOVA with Greenhouse-Geisser correction. A blunted HVR was considered to be a <10% increase in minute ventilation. Post hoc analysis was conducted with Bonferroni adjustment. Factors associated with the magnitude of HVR were assessed using Spearman correlation. Significance was considered at *p* < 0.05. Data analysis was performed with the use of IBM Statistical Package for the Social Science (SPSS) software (IBM Corp., Chicago, Ill., USA; version 23).

## Results

### Study population

An oxygen reduction test was completed in 39 infants surviving very preterm birth at a mean (SD) of 14.4 (1.0) months corrected postnatal age. Sixteen of the 39 infants were classified as having BPD. As expected, infants with BPD were more immature, had lower birthweights and required increased respiratory support compared to those infants without BPD. Demographic and neonatal information are shown in Table 1.

**Table 1:** Describing the study population of preterm born infants, and by neonatal classification of bronchopulmonary dysplasia (BPD).

	All Preterm	No BPD	BPD
N	39	23	16
Male/Female (% Male)	30/9 (76.9)	17/6 (73.9)	13/3 (81.3)
Gestational, (w)	27.6 (25.0–29.1)	29.0 (27.7–30.0)	25.0 (24.0–26.3)^a^
BWt, (g)	920 (750–1130)	1,005 (830–1364)	765 (685–945)^a^
BWt z-score, mean (SD)	−0.06 (0.93)	−0.43 (0.85)	0.48 (0.78)^a^
Any respiratory support, (d)	47.2 (23.9–92.4)	31.3 (9.3–42.8)	94.0 (75.0–100.6)^a^
- MV, (d)	0.5 (0.3–9.2)	0.3 (0.0–0.6)	17.5 (1.4–36.8)^a^
- CPAP, (d)	34.5 (7.0–50.9)	15.8 (5.4–34.5)	51.5 (45.1–57.9)^a^
- HHF, (d)	12.5 (4.6–19.9)	9.7 (1.7–13.2)	19.9 (11.8–31.7)^a^
Supp. O_2_ duration, (d)	6.7 (0.6–68.1)	0.9 (0.0–4.2)	80.2 (52.9–122.6)^a^
Caffeine duration, days	48.5 (27.8–67.3)	36.5 (20.0–48.3)	69.5 (60.3–86.5)^a^
cPNA, mean (SD) months	14.4 (1.0)	14.3 (1.1)	14.5 (1.0)
Wt, mean (SD) kg	9.9 (1.5)	9.6 (1.2)	10.3 (1.8)

All data are presented as median (IQR) unless otherwise indicated.

^a^ Indicates a significant difference between the preterm groups with and without BPD (*p* < 0.05). BWt, birthweight; MV, mechanical ventilation; CPAP, continuous positive airway pressure; HHF, humidified high flow; cPNA corrected postnatal age at study; Wt, weight at study.

### Hypoxic ventilatory response in infants born preterm at 12–15 months corrected postnatal age

**Figure 2:**
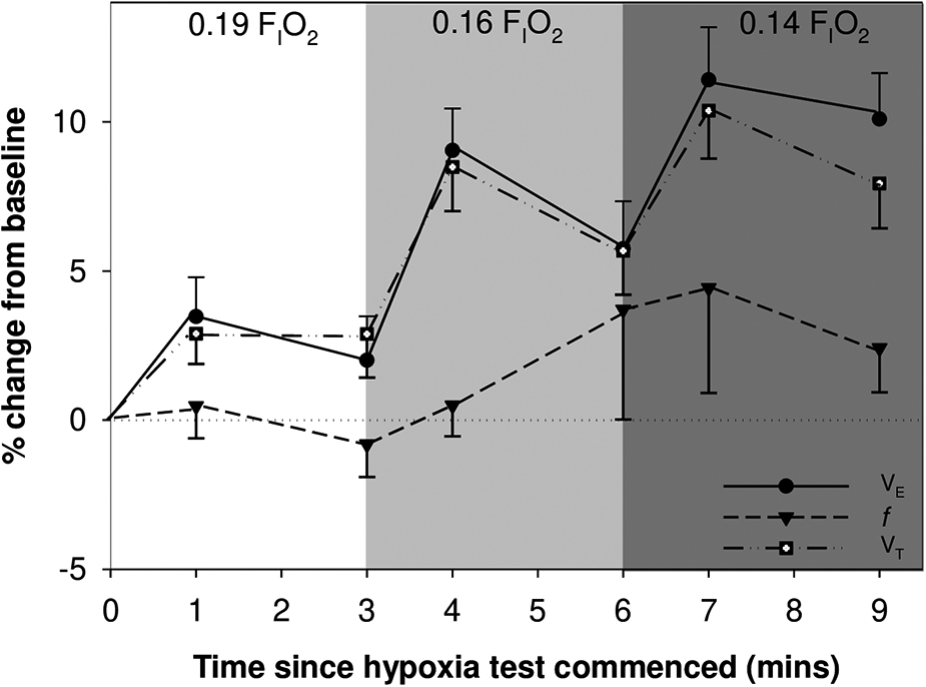
Mean (SEM) change in minute ventilation (*V*_E_), tidal volume (*V*_T_) and respiratory rate (*f*) are shown over time during the hypoxia challenge.

Infants born very preterm demonstrated a HVR: there was a mean (SD) increase in *V*_E_ of 11.4 (10.7) % in response to decreasing F_I_O_2_ from 0.21 to 0.14 (Figure 2). However, 17 infants (44%) failed to increase *V*_E_ by ≥10% (range −14% to 9%) and were considered to have a blunted response to hypoxia, compared to the responders (range 10% to 37%). Of these 17 infants, 5 had reductions in *V*_E_ that was below baseline values; all were boys.

We determined that mean *V*_T_, *V*_E_, *V*_T_/*t*_TOT,_ *V*_T_/*t*_I,_ and area of the flow volume loop differed significantly between time points during the exposure to moderate hypoxic gas mixture (*p* < 0.001): Each of these outcome variables increased during the first minute of exposure to 0.16 F_I_O_2_, and during both minute 1 and 3 of 0.14 F_I_O_2_, compared to baseline (See Table 2 for all comparisons).

**Table 2:** Tidal breathing measures at baseline and during hypoxic conditions.

	0.21 F_I_O_2_	0.19 F_I_O_2_		0.16 F_I_O_2_		0.14 F_I_O_2_		*p*-value
	Baseline*	Minute 1^a^	Minute 3^b^	Minute 1^c^	Minute 3^d^	Minute 1^e^	Minute 3^f^	
*t*_I_ (s)	0.9 (0.1)	0.9 (0.2)	0.9 (0.1)	0.9 (0.2)	0.9 (0.2)	0.9 (0.2)	0.9 (0.2)	0.510
% change from baseline	–	1.0 (10.9)	1.1 (10.4)	1.1 (10.0)	0.7 (10.9)	0.8 (13.4)	−1.0 (12.2)	
*t*_E_ (s)	1.3 (0.3)	1.3 (0.2)	1.3 (0.2)	1.3 (0.2)	1.3 (0.3)	1.2 (0.2)	1.3 (0.3)	0.265
% change from baseline	–	−1.2 (6.7)	1.2 (7.1)	−1.2 (7.2)	−0.5 (8.1)	−1.7 (9.8)	−1.2 (12.3)	
*t*_PTEF_ (s)	0.3 (0.1)	0.3 (0.1)	0.3 (0.2)	0.3 (0.1)	0.3 (0.1)	0.3 (0.1)	0.3 (0.2)	0.191
% change from baseline	–	5.3 (18.5)	7.9 (30.6)	1.0 (16.5)	2.2 (19.8)	7.6 (26.3)	10.5 (30.7)	
*V*_T_ (ml.kg^−1^)	8.4 (1.4)^c,e,f^	8.7 (1.6)^c,d,e,f^	8.6 (1.4)^c,e,f^	9.1 (1.6)*^,a,b^	8.8 (1.6)^a,e^	9.2 (1.6)*^,^^a,b,d^	8.9 (1.4)*^a^^,^^b^	***p* < 0.001**
% change from baseline	–	2.9 (6.2)	2.9 (8.8)	8.5 (8.7)	5.7 (8.6)	10.4 (9.8)	7.9 (9.0)	
RR min^−1^	28.5 (4.4)	28.8 (4.8)	28.3 (4.6)	28.7 (4.9)	29.3 (5.7)	29.5 (5.2)	29.3 (5.1)	0.229
% change from baseline	–	0.5 (6.6)	−0.8 (6.6)	0.5 (6.1)	3.7 (21.5)	4.4 (21.4)	2.4 (8.9)	
*V*_E_ (ml.min^−1^.kg^−1^)	236 (35)^c,e,f^	245 (41)^c,e,f^	241 (38)^c,d,e,f^	257 (45)*^,^^a^^,^^b^	247 (42)^b,e,f^	261 (42)*^,^^a,b,d^	257 (42)*^,^^a,b,d^	***p* < 0.001**
% change from baseline	–	3.5 (8.0)	2.0 (8.9)	9.0 (8.3)	5.7 (9.3)	11.4 (10.7)	10.1 (9.3)	
*t*_I_/*t*_TOT_	41.2 (5.2)	41.6 (5.6)	41.1 (4.8)	42.0 (4.7)	41.9 (5.5)	41.9 (5.5)	41.6 (5.3)	0.521
% change from baseline	–	1.0 (6.5)	−0.2 (5.4)	1.3 (6.8)	0.7 (7.8)	1.4 (8.2)	0.5 (6.4)	
*V*_T_/*t*_TOT_ (ml.s^−1^)	38.2 (5.0)^c,d,e,f^	39.6 (5.8)^c,e,f^	38.9 (5.4)^c,d,e,f^	41.6 (5.5)*^,^^a,b^	40.4 (5.4)*^,^^b,e,f^	42.7 (5.4)*^,^^a,b,d^	41.8 (5.1)*^,^^a,b^	***p* < 0.001**
% change from baseline	–	3.7 (8.2)	2.2 (8.8)	9.2 (8.3)	6.1 (9.3)	11.9 (11.0)	9.9 (9.9)	
*V*_T_/*t*_I_ (ml.s^−1^)	94.2 (16.0)^c,d,e,f^	97.0 (18.5)^e,f^	95.3 (15.1)^c,e,f^	100.0 (16.0)*^,^^b^	97.9 (16.1)^e^	103.5 (17.2)*^,^^a,b,d^	102.4 (15.9)*^,^^a,b^	***p* < 0.001**
% change from baseline	–	2.9 (11.2)	2.6 (12.3)	8.2 (12.3)	6.0 (13.5)	10.7 (13.0)	10.1 (12.0)	
AFVL	15,427 (3589)^c,e,f^	16,241 (4173)^c,e,f^	15,840 (3734)^c,e,f^	17,571 (4345)*^,^^a,b^	16,921 (4178)^e^	18,568 (4796)*^,^^a,b,d^	17,697 (4008)*^,^^a,b^	***p* < 0.001**
% change from baseline	–	5.7 (14.7)	4.8 (18.9)	15.9 (19.8)	11.6 (21.6)	20.7 (21.1)	17.1 (17.5)	

Tidal breathing measures at baseline and during hypoxic conditions. Data are presented as mean (SD).

*indicates significantly different to baseline.

^a,b,c,d,e,f^ Indicate a significant difference from the corresponding timepoint during the hypoxia challenge (as indicated in the header). *t*_I_, inspiratory time; *t*_E_, expiratory time, *t*_PTEF_, time to peak tidal expiratory flow; *V*_T_, tidal volume; RR, respiratory rate; VE, minute ventilation; *t*_TOT_, total cycle time; AFVL, area under flow volume loop. % change is % change from baseline. N.B Means of whole group presented - analysis included only those infants with all timepoints (*N* = 31).

### Factors associated with a blunted ventilatory response to hypoxia

**Figure 3:**
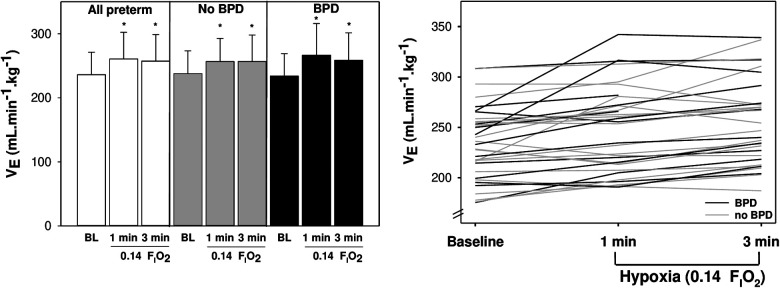
Mean ± SD (left) and individual (right) minute ventilation (*V*_E_) is shown at baseline and during 14% O_2_ for preterm infants with (black) and without (grey) bronchopulmonary dysplasia. *indicates different from baseline (*p* < 0.05).

As the first minute of exposure to an F_I_O_2_ of 0.14 elicited the strongest ventilatory response to hypoxia, we tested associations between the magnitude of the HVR and neonatal factors at this timepoint to explore if there were any predictors of HVR after preterm birth. The HVR was generally not different between preterm infants with or without BPD (see Figure 3 for example). The exception to this statement is that infants with BPD had decreased *Δt*_I_ (mean difference = −9.9%; 95% CI = −17.7, −2.2; *p* = 0.013) during the first minute of 0.14 F_I_O_2._ Accordingly, we also observed decreased Δ*t*_I_/*t*_TOT_ (−5.4%; −10.6, −0.3; *p* = 0.039) and increased Δ*V*_T_/T_I_ (10.7%; 2.9, 18.4; *p* = 0.009) in those with BPD.

**Figure 4:**
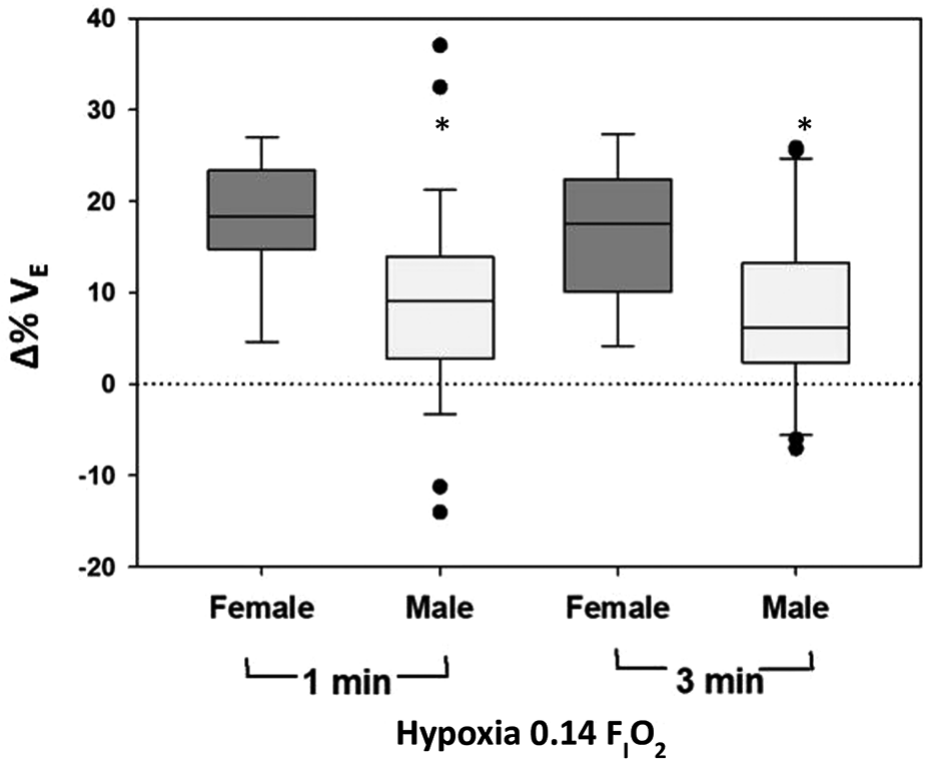
Change (% from baseline) in minute ventilation (*V*_E_) during hypoxia is shown as a function of sex.

Male infants had a reduced HVR, with decreased Δ*V*_E_ (−9.1%; −15.4, −2.8; *p* = 0.007), Δ*V*_T_/*t*_I_ (−11.0%; −20.3, −1.8; *p* = 0.025), and Δ*V*_T_/*t*_TOT_ (−9.6%; −16.4, −2.8; *p* = 0.008) in response to 0.14 F_I_O_2_ compared to female infants (Figure 4).

**Figure 5:**
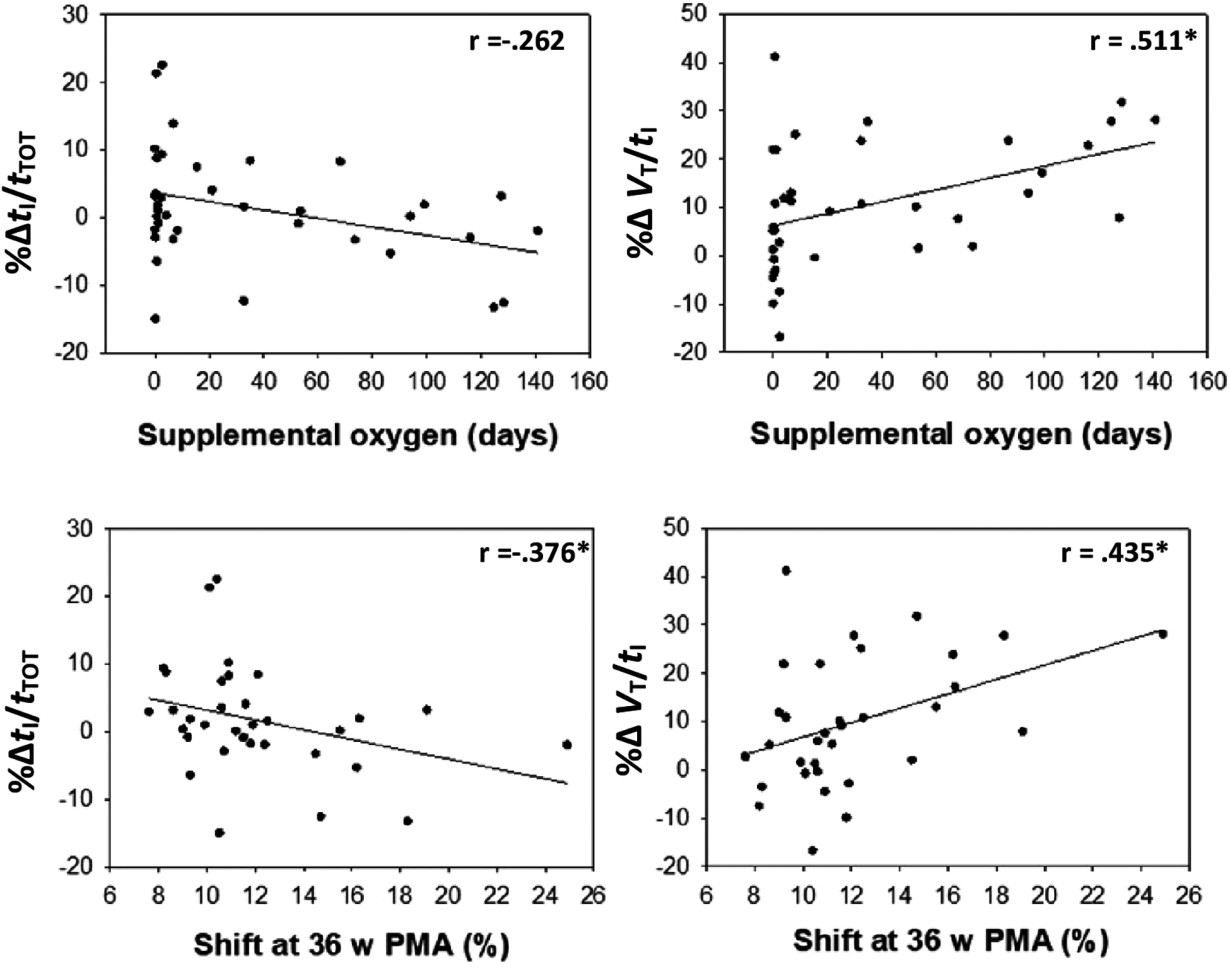
Change (% from baseline) in the driving (*V*_T_/*t*_I_) and timing (*t*_I_/*t*_TOT_) components of the breathing cycle during hypoxia are correlated with days of supplemental oxygen and shift at 36 weeks’ PMA.

Several neonatal factors were associated with HVR magnitude including gestational age, duration of respiratory support, duration of oxygen therapy, and shift of the oxyhaemoglobin curve at 36 weeks postmenstrual age (Supplementary Table S1; Figure 5). The *Δ*HVR was not associated with the *Δ*SpO_2_ (*p* > 0.05).

### Discussion

We report that infants born very preterm have a small ventilatory response to hypoxia at 12–15 months corrected postnatal age (mean (SD):11.4% (10.7) increase in minute ventilation), that is largely driven by an increase in tidal volume. However, the HVR was blunted (<10% increase in minute ventilation) in almost half of the infants studied. Interestingly, the magnitude of the HVR was not reduced in infants with BPD but was reduced in male infants.

The magnitude of the HVR varies considerably in healthy individuals at sea level, including with age. For example, the HVR is small in newborns and very young infants (∼15% at 3 months of age), when exposed to 0.15 F_I_O_2_ (18). In contrast, healthy adults can increase minute ventilation by up to ∼60% in the first five minutes after exposure to hypoxia (measured by an arterial oxygen saturation of 80%), remaining at 26% above baseline 25 min after commencing hypoxic exposure (10). Unfortunately, there are limited HVR data in healthy young children and none for infants in the second year of life (to our knowledge). Further, the methods of reporting the available data in children hinder direct comparison to the % change mentioned above for infants and adults. These previous data in healthy populations do however show that children have an increased HVR compared to adults (when corrected for weight) (30), with the HVR declining throughout adolescence and adulthood (30). Taken together, and in the absense of term born controls, we proposed an *a priori* cut off of <10% increase *V*_E_ to constitute a blunted response to hypoxia; a HVR 1/3 lower than ∼3 month old infants. Indeed, without control data to support the selected cut off, our analysis of the data does potentially underestimate the proportion of infants displaying a blunted HVR in the second year of life. Direct comparison of our findings with earlier studies in infants, children and adults is also hampered by the differing methods of application of hypoxia, with some previous studies using headbox methods (slow change in inspired O_2_) while others have used an instant reduction in F_I_O_2_ from baseline (15, 17, 22), rather than the stepwise approach to reduction in F_I_O_2_ utilised in our study. The stepwise approach, compared with the intantaneous reduction method, may elicit a different magnitude of response, and should be considered as a limitation when comparing these data to those obtained from the “gold standard” steady state assessment of the HVR. Regardless, our findings highlight that further investigation on the maturation of the HVR beyond the first year of life, particularly in the preterm infant, is warranted.

A single small study has previously evaluated the ventilatory response to hypoxia beyond the first year of life, and reported a blunted HVR in young adults born <32 weeks gestation compared to term born young adults (receiving a 60 s hypoxic exposure), despite similar respiratory function and exercise capacity (31). Unfortunately, the sample size of 13 individuals prevented any exploration of factors potentially contributing to a blunted HVR. However, the observation of blunted HVR in survivors of very preterm birth is concerning and may pose additional lifetime risk of disordered breathing during sleep and in response to challenges such as high altitude or during anaesthesia.

The ventilatory response to hypoxia (% change in *V*_E_) was not different between infants with and without BPD, nor associated with NICU factors such as the duration of supplemental oxygen. However, the strategies by which the infants altered their ventilation was related to neonatal factors. For example, infants with higher duration of supplemental O_2,_ or increased right shift of the oxyhaemoglobin dissociation curve in the NICU, showed increase in the “driving components” (*V*_T_/*t*_I_) of the breathing cycle and decreases in the “timing components” or duty cycle (*t*_I_/*t*_TOT_) during hypoxia, largely influenced by a reduced inspiratory time. Such observations may highlight ongoing alterations to central respiratory neural networks and/or respiratory system compliance in preterm infants requiring additional respiratory support in the NICU. Indeed, a short inspiratory time (*t*_I_) is common in preterm neonates, relative to term born infants, and hinders adequate ventilation and oxygenation (32). Further, children with increased duration of supplemental oxygen have “stiffer” lungs during childhood (33) and an altered ventilatory response to exercise (34). It remains likely that the altered HVR of preterm infants with lung disease involves a complex interaction between immaturity and injury of the peripheral chemoreceptors, central control of breathing, and respiratory mechanics (35).

We showed a reduced ventilatory response to hypoxia in males born preterm. Further, all 5 infants with *V*_E_ falling below baseline levels in response to hypoxia were boys. Sex differences in the magnitude of the acute HVR have generally not been observed in term-born humans, once accounting for differences in body size (30) or baseline minute ventilation (36). However, preclinical studies suggest that there are critical developmental windows or “sensitive periods” where exposure to hypoxia or hyperoxia can have lifelong impacts on the control of breathing (37–39), and that these impacts can differ between males and females (40). For example, neonatal hypoxia impairs the HVR in adult male, but not adult female, rats (39). Developmental sensitive periods have not been identified in human infants. However, the heterogeneity of HVR in this study may support the idea, and males may be disproportionately affected by long term disturbances in the control of breathing. Indeed the prevalence of infant respiratory distress syndrome, sudden infant death syndrome (SIDS) and obstructive sleep apnoea (OSA) is higher in males than females (41–43) in the term born population. Similarly, the risk of SIDS (44), brief resolved unexplained events (BRUE) (45), and sleep disordered breathing (46) is increased in those surviving preterm birth and is inversely correlated to gestational age at birth. Together, these findings suggest that males born preterm may warrant further screening for disorders of respiratory control. Our findings at one year of age indicate that the risk of hypoxia related morbidity and mortality is not isolated to early infancy.

Our infants were studied while sedated with chlroral hydrate, which likely affected sleep state. Chloral hydrate is reported as safe to use during hypoxia and has minimal effect on ventilation (47, 48). However, in adults, sleep state influences ventilatory response to hypoxia, with the HVR being smaller in rapid eye movement (REM) sleep compared with quiet sleep (49). In contrast, sleep state has no effect on the HVR in infants: Richardson et al., show no difference in the HVR between healthy term infants tested in active sleep compared to quiet sleep at 5–6 months of age (18). Hence, the influence of sleep state on the HVR may be the result of maturation.

In summary, we show that some infants surviving very preterm birth have an attenuated ventilatory response to hypoxia that persists into the second year of life, especially males. Although it is unclear why some preterm infants have a dampened HVR, these findings further our understanding that preterm infants may be at increased risk of disorders of respiratory control throughout life.

## Data Availability

The original contributions presented in the study are included in the article/Supplementary Material, further inquiries can be directed to the corresponding author/s.
