# Supplementary material for: The ventilatory response to hypoxia is blunted in some preterm infants during the second year of life
Source: Front Pediatr. 2022 Oct 26;10:974643. doi: 10.3389/fped.2022.974643 (PMC9661422; doi:10.3389/fped.2022.974643)
Supplement: Supplementary file 1 [file Table1.docx]

**Supplementary table 1:** Non-parametric correlations to assess the relationship between demographic and neonatal factors, and the magnitude of the hypoxic ventilatory response. * represents p<0.05. ** represents p<0.001. BWt, birth weight; Wt, weight; MV, mechanical ventilation; CPAP, continuous positive airway pressure; HHF humidified high flow; Shift – rightward shift of the Peripheral oxyhaemoglobin vs inspired partial pressure of oxygen dissociation curve; SpO2, peripheral oxyhaemoglobin saturation.

|  | *t*_I_  (% change) | *t*_E_  (% change) | *t*PTEF  (% change) | *V*_T_/kg  (% change) | RR  (% change) | *t*_I_/*t*_TOT_  (% change) | *V*_T_/*t*_TOT_  (% change) | *V*_T_/*t*_I_  (% change) | *V*_E_/kg  (% change) | AFVL  (% change) |
| --- | --- | --- | --- | --- | --- | --- | --- | --- | --- | --- |
| Gestational age, w | **.362^*^** | 0.183 | **.432^**^** | 0.095 | **-.431^**^** | 0.173 | -0.225 | **-.348^*^** | -0.206 | -0.082 |
| BWt z-score | 0.023 | 0.310 | -0.094 | -0.006 | -0.068 | -0.116 | -0.154 | -0.020 | -0.163 | 0.089 |
| MV duration, h | **-.367^*^** | 0.112 | -0.291 | -0.220 | 0.270 | **-.352^*^** | -0.022 | 0.290 | -0.026 | -0.033 |
| CPAP duration, h | -0.159 | -0.076 | **-.330^*^** | 0.070 | 0.241 | -0.117 | 0.219 | 0.321 | 0.210 | 0.139 |
| HHF duration, h | -0.050 | -0.110 | **-.398^*^** | -0.138 | 0.219 | 0.144 | -0.094 | -0.091 | -0.099 | -0.161 |
| O_2_, d | **-.333^*^** | -0.104 | **-.493^**^** | 0.037 | 0.318 | -0.262 | 0.316 | **.511^**^** | 0.315 | 0.225 |
| Highest [O_2_] administered (%) | -0.251 | 0.006 | -0.176 | -0.046 | 0.199 | -0.284 | 0.109 | 0.307 | 0.093 | 0.027 |
| Caffeine duration, d | -0.256 | -0.088 | -0.057 | 0.004 | 0.266 | -0.156 | 0.159 | 0.267 | 0.159 | 0.046 |
| Cord blood pCO2, mmHg | -0.070 | -0.068 | 0.137 | -0.233 | -0.003 | -0.005 | -0.185 | -0.159 | -0.195 | -0.320 |
| Shift at 36 weeks PMA, mmHg | -0.222 | 0.098 | **-.367^*^** | 0.054 | 0.120 | **-.376^*^** | 0.253 | **.435^*^** | 0.270 | 0.253 |
| Wt at study kg | **.372^*^** | 0.209 | 0.243 | 0.147 | -0.279 | 0.114 | -0.166 | -0.202 | -0.157 | 0.022 |
| SpO_2_ at baseline (FiO_2_ = 0.21) | -0.175 | -0.057 | 0.092 | 0.027 | 0.051 | -0.071 | 0.049 | 0.131 | 0.068 | 0.068 |
| SpO_2_ at FiO_2_= 0.14 | 0.089 | -0.073 | 0.095 | 0.041 | 0.034 | 0.084 | -0.037 | -0.055 | -0.017 | -0.073 |
